# Supplementary material for: Genome-Wide Association Studies of Body Weight and Average Daily Gain in Chinese Dongliao Black Pigs
Source: Int J Mol Sci. 2025 Apr 7;26(7):3453. doi: 10.3390/ijms26073453 (PMC11989284; doi:10.3390/ijms26073453)
Supplement: Supplementary file 1 [file ijms-26-03453-s001.zip › ijms-3537031-supplementary.pdf]

# Supplementary Figures

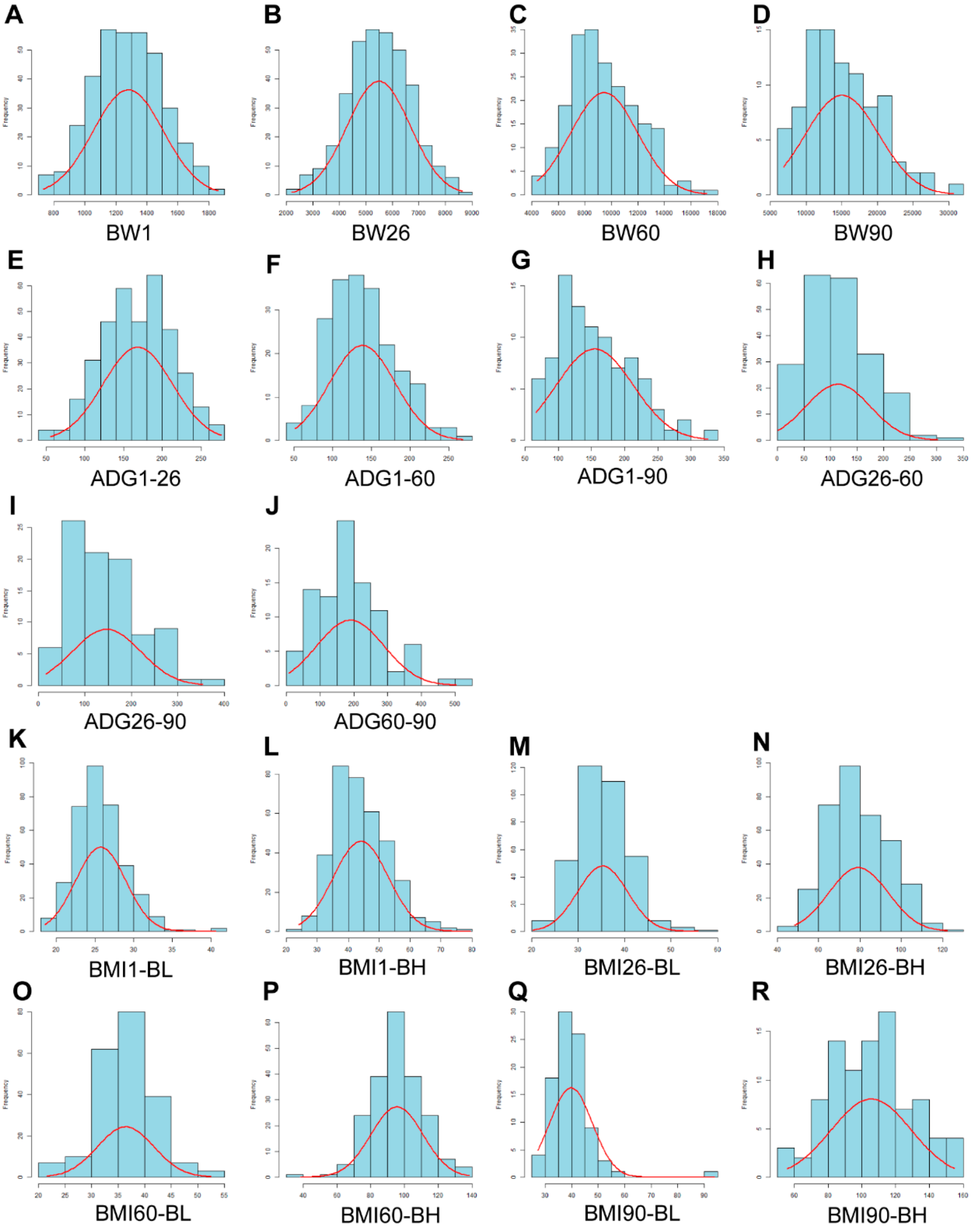


**Figure S1.** Frequency distribution histogram for 18 growth traits.

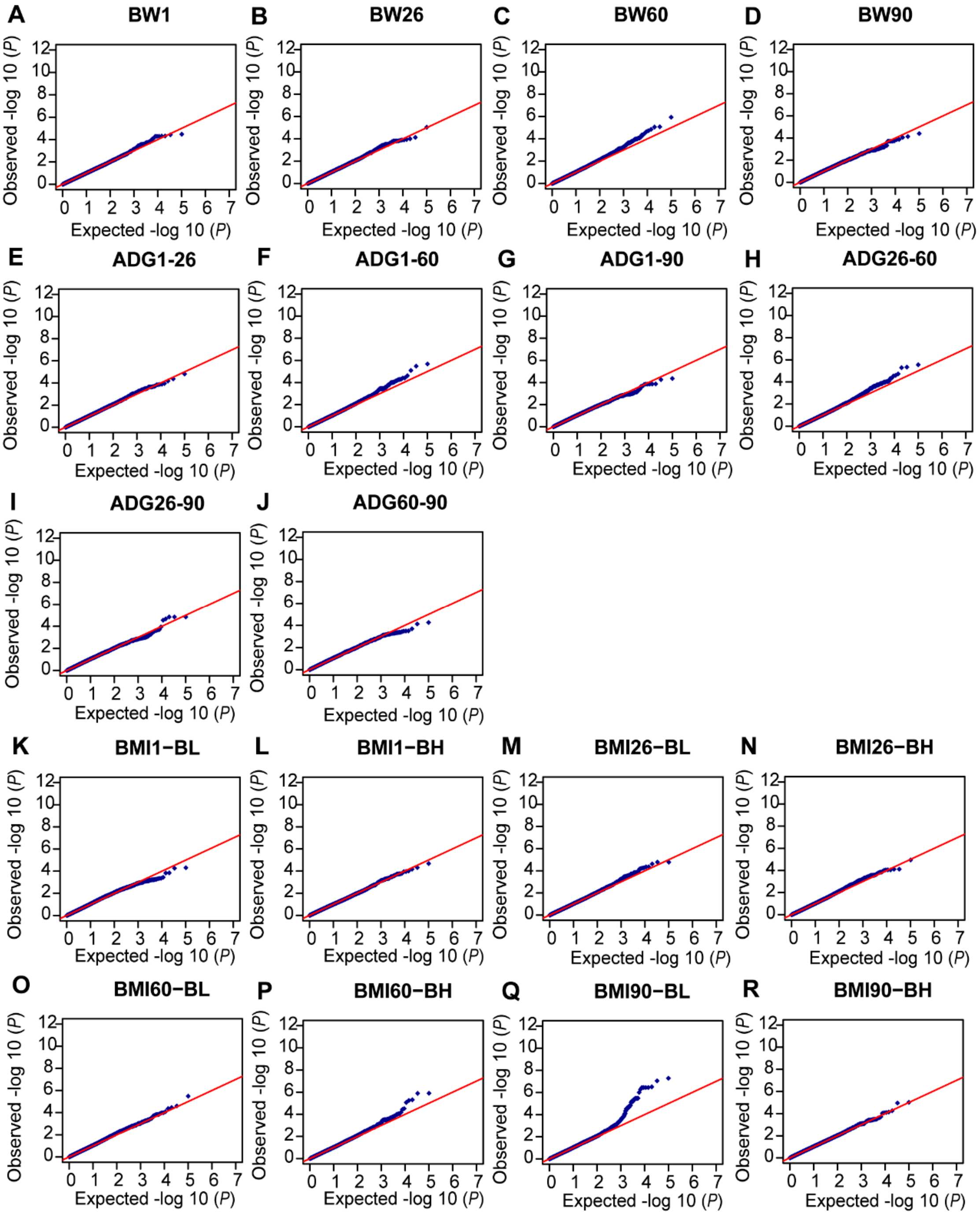


**Figure S2.** The quantile-quantile plots show the observed distribution of *P*-values against the expected *P*-values under the null hypothesis of no association. The horizontal axis shows −log10 transformed expected *P*-values, and the vertical axis indicates −log10 transformed observed P values.

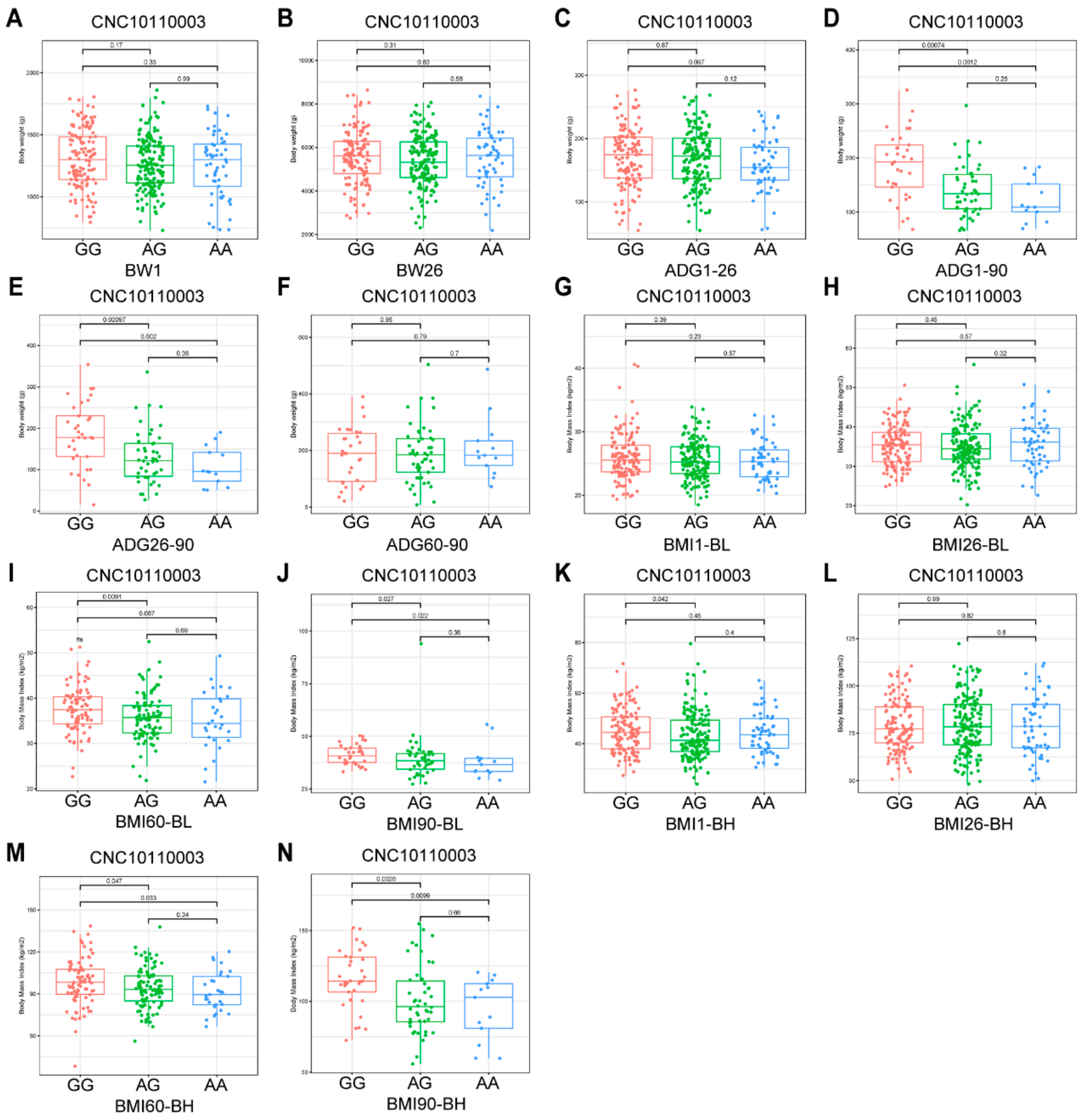


**Figure S3.** The box plot illustrates the discrepancies in the three genotype frequencies between body weight, mean daily weight gain, and body mass index for the GWAS results (CNC10110003).
